# Rapid identification of *Aconitum* plants based on loop-mediated isothermal amplification assay

**DOI:** 10.1186/s13104-019-4463-1

**Published:** 2019-07-15

**Authors:** Masashi Kitamura, Akira Kazato, Tadashi Yamamuro, Hirokazu Ando, Yohei Sasaki, Ryuichiro Suzuki, Yoshiaki Shirataki

**Affiliations:** 1 Laboratory of Pharmacognocy and Natural Medicines, Department of Pharmaceutical Sciences, Faculty of Pharmacy and Pharmaceutical Sciences, Josai University, 1-1, Keyakidai, Sakado, Saitama 350–0295 Japan; 2 Laboratory of Molecular Pharmacognosy, Division of Pharmaceutical Sciences, Graduate School of Medical Sciences, Kanazawa University, Kakuma-machi, Kanazawa, Ishikawa 920-1192 Japan; 3 Forensic Science Laboratory, Ishikawa Prefectural Police H.Q, 1-1 Kuratsuki, Kanazawa, Ishikawa 920-8553 Japan; 4 National Research Institute of Police Science, 6-3-1 Kashiwanoha, Kashiwa, Chiba 277-0882 Japan

**Keywords:** *Aconitum*, Isothermal amplification, LAMP, Accidental ingestion, Toxic plants

## Abstract

**Objective:**

*Aconitum* plants (Ranunculaceae) exhibit toxicity, and accidental ingestion of the plants has been reported in Japan. Identifying the cause of poisoning is important for emergency medical treatment, and a rapid and simple detection technique is required for the identification of poisoning cause. In the present study, we developed a rapid and simple method for detecting *Aconitum* plant DNA using a loop-mediated isothermal amplification (LAMP) assay.

**Results:**

Specific LAMP primers for *Aconitum* plants were designed based on the *trnL*–*trnF* intergenic spacer region. Using the LAMP primers, the LAMP assay included an initiation reaction of 10 min followed by amplification for 20 min at the isothermal reaction temperature of 65 °C. The LAMP reaction was demonstrated to be specific and highly sensitive to *Aconitum* plants, given that the assay can be used for 1 pg of purified DNA. Using raw extracted DNA as template, the entire detection procedure from DNA extraction to final detection required only 30 min. Moreover, the protocol identified samples containing approximately 5 mg of *Aconitum* plants cooked and digested with artificial gastric juice. The currently proposed protocol exhibits good potential as a screening method of *Aconitum* plant poisoning for emergency medical care.

**Electronic supplementary material:**

The online version of this article (10.1186/s13104-019-4463-1) contains supplementary material, which is available to authorized users.

## Introduction

In the case of accidental ingestion of toxic plants, rapid identification is important for emergency medical treatment. *Aconitum* plants, which belong to the family Ranunculaceae, comprise over 200 species worldwide, and approximately 20 species grow naturally in Japan [1–5]. The leaves of *Aconitum* plants are similar to those of edible plants, such as *Anemone flaccida* (Ranunculaceae) and *Parasenecio delphiniifolius* (Compositae), and often grow in the same habitat; therefore, accidental ingestion of *Aconitum* plants could occur [6]. *Aconitum* plants contain alkaloids, such as aconitine, which causes systemic paralysis, nausea, and vomiting, followed by dizziness, palpitation, hypotension, arrhythmia, shock, and coma [7, 8]. Some cases of poisoning lead to fatal accidents because of respiratory failure [6, 9–11]. In Japan, a total of 14 patients were reported to be poisoned by *Aconitum* plants from 2008 to 2017, out of which three patients died because of toxicity [12]. Toxic plants can be identified by morphological analysis or chemical analysis using high-performance liquid chromatography (HPLC) or liquid chromatography-mass spectrometry (LC/MS) [6, 9–11, 13]. Recently, DNA-based analytical methods, such as real time polymerase chain reaction (PCR), have been proposed for the detection of toxic plants, such as *Aconitum* plants and *Veratrum album* [14, 15]. However, these analytical methods require long processing time and a well-equipped laboratory. Therefore, a rapid and simple detection method is desirable to facilitate emergency medical treatment of poisoning. A loop-mediated isothermal amplification (LAMP) assay is a DNA amplification method performed under isothermal conditions at 60 °C to 65 °C; the amplification efficiency of the assay is 100- to 1000-fold higher than that of PCR [16]. The LAMP method can be performed within a short time using a simple device, such as a block incubator, and has consequently been widely used for nucleic acid detection of viruses, bacteria, and plants [17–19]. In the present study, we developed a rapid and simple LAMP-based method for the identification of *Aconitum* plants. Our protocol could be useful as a screening method for poisoning cases that are suspected to be caused by *Aconitum* plants in emergency medical care.

## Main text

### Materials and methods

#### Plant materials and DNA extraction

*Aconitum japonicum* Thunb. subsp. *subcuneatum* (Nakai) Kadota, *Aconitum sachalinense* F. Schmidt subsp. *yezoense* (Nakai) Kadota, *Aconitum chinense* Siebold ex Paxton, *Anemone flaccida*, and *Parasenecio delphiniifolius* were obtained from the medicinal botanical gardens of Kanazawa University and Josai University. A total of 24 plant samples used for specificity test were obtained from the medicinal botanical garden of Kanazawa University and are provided in Additional file 1. Total genomic DNA was extracted using a DNeasy Plant Mini Kit (Qiagen, Germany) or NucleoSpin Plant II (Takara, Japan) and quantified using a NanoDrop 1000 instrument (ThermoFisher Scientific, USA). Extracted DNA was diluted to 5.0 ng/µL and used as DNA template. Optimization of the LAMP assay was performed using 5.0 ng/µL *Ac. japonicum* subsp. *subcuneatum* DNA as positive control. LAMP sensitivity was evaluated using 10 ng, 1 ng, 100 pg, 10 pg, 1 pg, and 100 fg of purified *Ac. japonicum* subsp. *subcuneatum* genomic DNA as template. Samples were prepared by frying with vegetable oil for 1, 3, and 10 min or boiling in water for 5 and 10 min. Afterwards, samples were treated with or without artificial gastric juice for 2 h [15]. Artificial gastric juice 1 L contained 2.0 g of sodium chloride, 3.2 g of pepsin, and 7.0 mL of 35% HCl and diluted with water to a final volume of 1 L. Template DNA was prepared with extracted DNA using an Easy DNA Extraction Kit version 2 (Kaneka, Japan). Approximately 5 mg of leaf was digested with 100 µL of alkaline lysis solution (Solution A) and heated at 98 °C for 8 min. The heat-treated sample was mixed with 14 µL of neutralization solution (Solution B), and the tenfold-diluted lysate was used as DNA template.

#### Sequencing and LAMP primer design

PCR targeting the *trnL*–*trnF* intergenic spacer region was conducted at the final volume of 25 µL with the following components: 2.5 µL of 1× PCR buffer (Takara), 0.2 mM each dNTP, 0.5 µM each of forward primer (5ʹ-3ʹ; CGA AAT CGG TAG ACG CTA CG) and reverse primer (5ʹ-3ʹ; ATT TGA ACT GGT GAC ACG AG), 0.625 U of Ex Taq DNA polymerase (TaKaRa), and 1 µL of genomic plant DNA. The PCR products were purified and sequenced using BigDye Terminator v1.1 (Applied Biosystems, USA) on a 3130xl DNA genetic analyzer or 310 DNA genetic analyzer (Applied Biosystems) following the manufacturer’s protocol. Obtained sequences of *Ac. japonicum* subsp. *subcuneatum*, *Anemone flaccida*, and *Parasenecio delphiniifolius* were aligned, and primers specific to *Ac. japonicum* subsp. *subcuneatum* were designed using PrimerExplore v4 (https://primerexplorer.jp). A loop primer was designed manually to improve reaction efficiency [20]. The LAMP primers consisted of FIP (5ʹ-3ʹ; TGG GGG TAA AGC GAA CTT TTT ATG ACT TTT TAA ATC GTG AGG GT), BIP (5ʹ-3ʹ; TAA TCC TTT TTT CAG CGG TTC CAA TCC GAT CCA TTT GTG AGA), F3 (5ʹ-3ʹ; TTA TAG TAA GAG GAA AAT CCG TC), B3 (5ʹ-3ʹ; AAA CTT GTG ATA AAA GAG AAA CC), and a loop primer (5ʹ-3ʹ; TGG GGA TAG AGG GAC TTG A).

#### LAMP assay

The LAMP assay was carried out using an Isothermal amplification kit (Nippon Gene, Japan) with the total volume of 20 µL containing 10 µL of 2× reaction mix, 0.2 µM each F3 and B3 primers, 1.6 µM each FIP and BIP primers, 0.8 µM loop primer, and 1 µL of genomic DNA as template. Fluorescence data of the LAMP reaction were obtained using Smart Cycler (Cepheid, USA) or Step One Plus Real-Time PCR System (ThermoFisher Scientific, USA). The LAMP reaction was carried out for 20 min at the constant temperature of 65 °C. Fluorescence signals were collected at 15-s intervals. The cycle threshold value was determined and converted to the amplification time. The reactions were carried out in triplicates. Results were expressed as standard deviation (min ± SD).

### Results

#### Sequencing and primer design

First, we sequenced the intergenic spacer regions between the *trnL*–*trnF* of *Ac. japonicum* subsp. *subcuneatum*, *Ac. sachalinense* subsp. *yezoense, Ac. chinense, Anemone flaccida*, and *Parasenecio delphiniifolius*. In Japan, *Ac. japonicum* subsp. *subcuneatum* and *Ac. sachalinense* subsp. *yezoense* are is predominant, and *Ac. chinense* is cultivated as an ornamental plant. Sequencing analyses showed that the three *Aconitum* plants had the same sequences of the *trnL*–*trnF* regions. The sequence alignment of the *trnL*–*trnF* regions of *Ac. japonicum* subsp. *subcuneatum* (DDBJ/EMBL/GenBank database accession No. LC435033), *Anemone flaccida* (accession No. LC435034), and *Parasenecio delphiniifolius* (accession No. LC435035) were performed and we designed the *Ac. japonicum* subsp. *subcuneatum*-specific LAMP primer set (FIP, BIP, F3, and B3) and the loop primer to shorten the reaction time (Additional file 2).

#### LAMP reaction

**Fig. 1:**
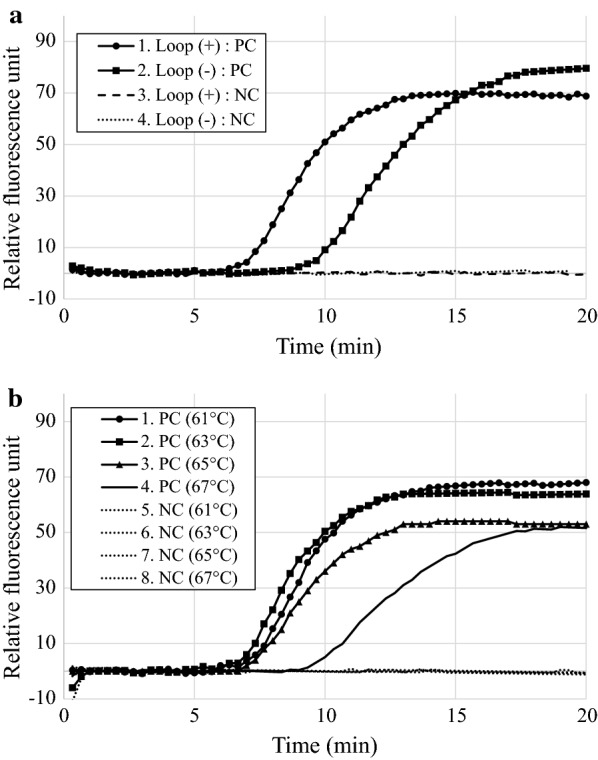
Optimization of the LAMP assay. **a** The LAMP assay was performed using the LAMP primer set with or without a loop primer. **b** The LAMP reaction was performed at 61, 63, 65, and 67 °C. Amplification profiles were obtained from positive control (PC) samples using *Aconitum. japonicum* subsp. *subcuneatum* DNA (5 ng) and negative control (NC) samples using *Anemone flaccida* DNA (5 ng)

Using the LAMP primer set, the LAMP assay was performed using *Ac. japonicum* subsp. *subcuneatum* DNA to optimize the LAMP conditions (Fig. 1). The LAMP reaction using the loop primer started at 7.2 ± 0.1 min, which was shorter than the reaction time without the loop primer (10.1 ± 0.39 min) (Fig. 1a). Next, the LAMP reaction was carried out at 61, 63, 65, and 67 °C. DNA amplification during the LAMP reaction at the incubation temperatures of 61, 63, and 65 °C was detected at approximately 7 min (7.5 ± 0.1, 7.1 ± 0.1, and 7.0 ± 0.2 min, respectively), whereas DNA amplification at 67 °C (9.5 ± 0.3 min) was slightly delayed (Fig. 1b). To evaluate the sensitivity of the LAMP assay, a serial tenfold dilution of purified DNA (10 ng, 1 ng, 100 pg, 10 pg, 1 pg, and 100 fg) was used as DNA template (Table 1). The DNA templates at 10 ng, 1 ng, 100 pg, 10 pg, and 1 pg showed successful amplification (6.8 ± 0.2, 8.0 ± 0.3, 8.9 ± 0.2, 10.3 ± 0.4, and 11.9 ± 0.3 min); however, amplification was not detected using 100 fg of DNA template. The sensitivity of the LAMP assay was higher than the previously reported sensitivity of real-time PCR (100 pg) [14]. We also confirmed that amplification of no-template controls did not occur for 20 min. To evaluate the specificity of the LAMP assay for *Aconitum* plants, the assay was conducted using three *Aconitum* species (*Ac. japonicum* subsp. *subcuneatum, Ac. sachalinense* subsp. *yezoense,* and *Ac. chinense*), *Anemone flaccida*, and *Parasenecio delphiniifolius*. *Ac. japonicum* subsp. *subcuneatum, Ac. sachalinense* subsp. *yezoense,* and *Ac. chinense* showed positive results from the LAMP assay (7.1 ± 0.1, 7.3 ± 0.0, and 7.2 ± 0.2 min, respectively), whereas *Anemone flaccida* and *Parasenecio delphiniifolius* tested negative. We further evaluated the results using 24 other randomly selected plant species to determine any false positive reaction (Additional file 1). No amplification was detected in the LAMP reactions with other plant species, except for *Coptis japonica*, which belongs to Ranunculaceae. However, amplification of *C. japonica* was observed in 2 of 5 replicates and the initiation time of amplification was approximately 18 min (17.8 min and 19.3 min), which was markedly longer than that of *Aconitum* plants (approximately 7 min) (Additional file 1). When the mixture containing 50 pg *Ac. japonicum* subsp. *subcuneatum* DNA and 50 ng *Anemone flaccida* DNA was used as template, amplification was observed at 9.8 ± 0.3 min. Therefore, we concluded that the LAMP primer set is specific to *Aconitum* plants.

**Table 1:** Results of LAMP detection assay

	Sample	DNA template	Reaction time^a^
Sensitivity	*Ac. japonicum* subsp. *subcuneatum*	10 ng	6.8 ± 0.2 min
		1 ng	8.0 ± 0.3 min
		100 pg	8.9 ± 0.2 min
		10 pg	10.3 ± 0.4 min
		1 pg	11.9 ± 0.3 min
		100 fg	nd
	(No template)		nd
Specificity	*Ac. japonicum* subsp. *subcuneatum*	5 ng	7.1 ± 0.1 min
	*Ac. sachalinense* subsp. *yezoense*	5 ng	7.3 ± 0.0 min
	*Ac. chinense*	5 ng	7.2 ± 0.2 min
	*Anemone flaccida*	5 ng	nd
	*Parasenecio delphiniifolius*	5 ng	nd
Mixture	*Ac. japonicum* subsp. *subcuneatum*	50 pg	10.0 ± 0.2 min
	*Ac. japonicum* subsp. *subcuneatum + Anemone flaccida*	50 pg + 50 ng	9.8 ± 0.3 min
	*Anemone flaccida*	50 ng	nd

*nd* not detected

^a^The reactions were carried out in triplacates

#### Simple protocol of LAMP and reactivity for processed samples

To simplify *Aconitum* plant detection, the LAMP assay was conducted using raw extracted DNA, which has been previously reported [19]. Processed samples were tested using the proposed protocol. Considering that *Anemone flaccida* and *Parasenecio delphiniifolius* are generally eaten after boiling or frying, *Ac. japonicum* subsp. *subcuneatum* leaves were boiled for 5 and 10 min or fried for 1, 3, and 10 min. Next, the processed samples were treated with artificial gastric juice at 2 h. Regardless of whether or not the samples were treated with artificial gastric juice, positive reactions were observed in the LAMP assay using non-processed samples, samples boiled for 5 and 10 min, and samples fried for 1 min and 3 min (Table 2). No amplification was observed using fried samples for 10 min probably because the DNA was degraded by excessive heating.

**Table 2:** Processed samples detected using the proposed protocol

	Non-processed sample (leaf)	Boiled sample		Fried sample		
		5 min	10 min	1 min	3 min	10 min
*Ac. japonicum* subsp. *subcuneatum*	+ (+)	+ (+)	+ (+)	+ (+)	+ (+)	− (−)
*Anemone flaccida*	− (−)	− (−)	− (−)	− (−)	− (−)	− (−)

Dried leaves were processed by boiling for 5 or 10 min or frying for 1, 3, and 10 min. The symbol ‘+’ indicates successful amplification within 20 min. No amplification within 20 min was denoted by the symbol ‘−’. The results of processed samples that were treated with artificial gastric juice for 2 h are shown in parentheses

### Discussion

In the present study, we developed a LAMP-based method for detecting *Aconitum* plant DNA. The LAMP primer set was designed based on the *trnL*–*trnF* intergenic spacer region of chloroplast DNA. The sequences of many *Aconitum* species are provided in the DDBJ/EMBL/GenBank databases. Out of 39 *Aconitum* species, 24 species had fully matched sequences with the LAMP primer binding sites, whereas the sequences of 15 species did not completely match the binding sites. Considering the diversity of *Aconitum* plants, which include over 200 *Aconitum* species worldwide, LAMP reactivity for each *Aconitum* species should be carefully evaluated. Using raw extracted DNA, the whole detection procedure from DNA extraction to detection was completed within approximately 30 min. Given that the protocol successfully identified most of the processed samples treated with artificial gastric juice, it can be used to screen unknown samples that are not identifiable by morphological analysis. It could test vomit samples from a patient, samples of a suspicious food that a patient ate, and samples from a suspicious plant. In the present study, we performed the LAMP assay on a real-time PCR device to monitor the amplification signals. However, the LAMP assay can be performed on a block incubator, and amplification can be checked by visual inspection (Additional file 3). Therefore, the protocol is expected to be useful as a screening method for poisoning cases that are suspected to be caused by *Aconitum* plants in emergency medical care. In summary, we developed a rapid and simple method for the detection of *Aconitum* plants based on a LAMP assay, a nucleic acid detection method. Our method showed high sensitivity, and the whole detection procedure can be completed within 30 min. Therefore, the proposed LAMP method could be useful as a screening method for poisoning in emergency medical treatment.

## Limitations

Considering that *Aconitum* plants comprise more than 200 species and several *Aconitum* plants have varieties and subspecies, LAMP reactivity for each *Aconitum* species should be evaluated in a careful manner.

## Additional files

**Additional file 1.** Plant information and LAMP Specificity for 24 randomly selected samples.

**Additional file 2.** Primer design for the LAMP assay.

**Additional file 3.** Sample tubes after LAMP reaction.

## Data Availability

The datasets used and/or analyzed in the current study are described in the manuscript. Any additional details will be made available from the corresponding author upon reasonable request.

## Abbreviations

bp: base pair
DNA: deoxyribonucleic acid
dNTP: deoxyribonucleotide triphosphate
LAMP: loop-mediated isothermal amplification
PCR: polymerase chain reaction
