# Supplementary material for: Rapid identification of Aconitum plants based on loop-mediated isothermal amplification assay
Source: BMC Res Notes. 2019 Jul 15;12:408. doi: 10.1186/s13104-019-4463-1 (PMC6631447; doi:10.1186/s13104-019-4463-1)
Supplement: Supplementary file 1 — Additional file 1. Plant information and LAMP Specificity for 24 randomly selected samples. [file 13104_2019_4463_MOESM1_ESM.docx]

**Plant information and LAMP Specificity for 24 randomly selected samples.**
