# Supplementary material for: Rapid identification of Aconitum plants based on loop-mediated isothermal amplification assay
Source: BMC Res Notes. 2019 Jul 15;12:408. doi: 10.1186/s13104-019-4463-1 (PMC6631447; doi:10.1186/s13104-019-4463-1)

**Primer design for the LAMP assay.**

Sequences of *Aconitum. japonicum* subsp. *subcuneatum* (DDBJ/EMBL/GenBank database accession No. LC435033), *Anemone flaccida* (accession No. LC435034), and *Parasenecio delphiniifolius* (accession No. LC435035) were aligned, LAMP primers specific for *Aconitum* plants were designed.


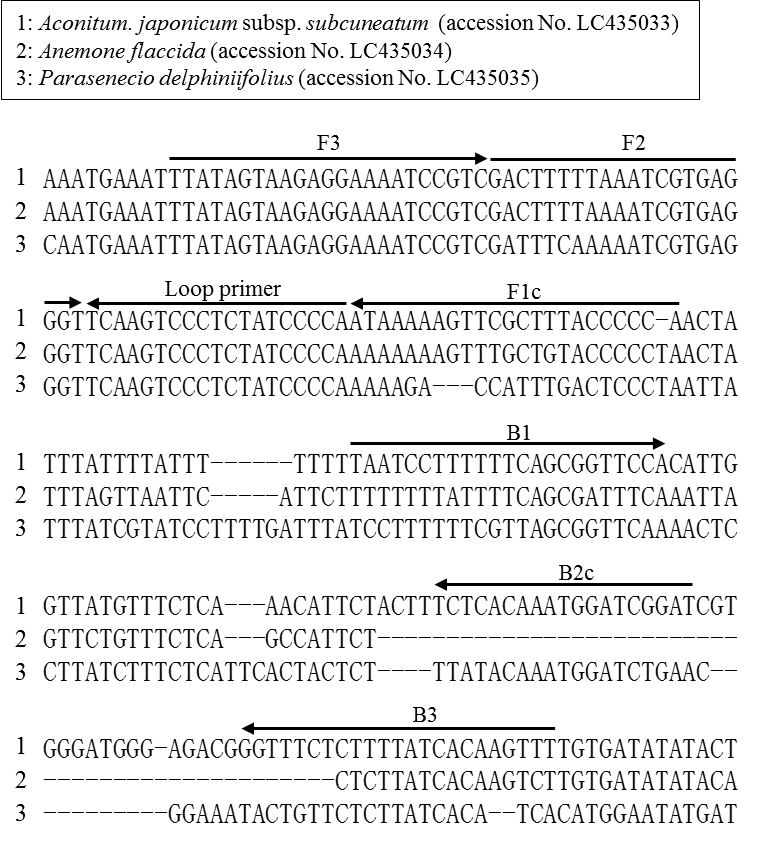


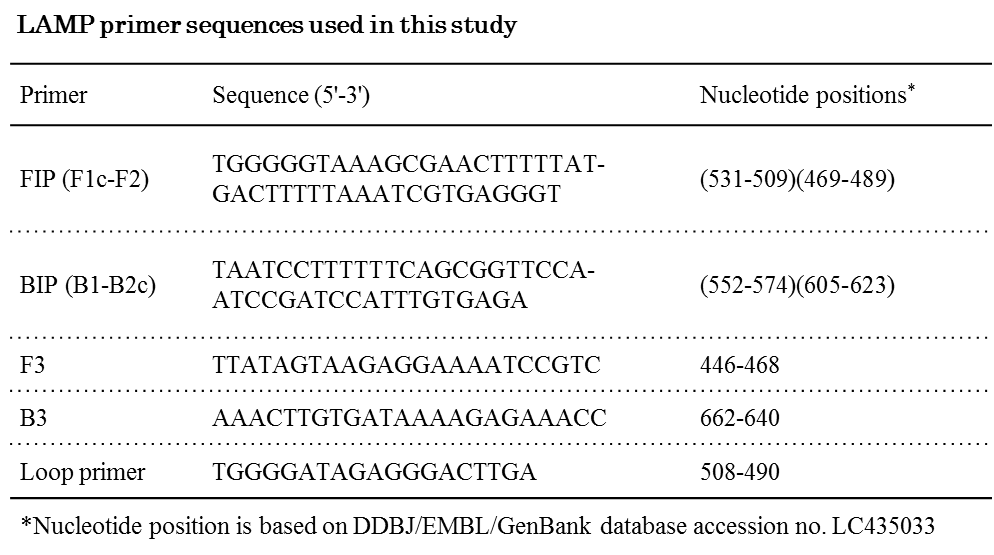

Supplement: Supplementary file 2 — Additional file 2. Primer design for the LAMP assay. [file 13104_2019_4463_MOESM2_ESM.docx]
