# Supplementary material for: Rapid identification of Aconitum plants based on loop-mediated isothermal amplification assay
Source: BMC Res Notes. 2019 Jul 15;12:408. doi: 10.1186/s13104-019-4463-1 (PMC6631447; doi:10.1186/s13104-019-4463-1)

**Sample tubes after LAMP reaction.** Simplified DNA detection protocol based on the LAMP assay was performed using the LAMP reaction mixture containing final concentration of 5*µ*M SYTO™ 16 Green Fluorescent Nucleic Acid Stain (ThermoFisher Scientific). The LAMP reaction was conducted at 63 °C for 20 min in a heating block and amplification was observed with a LED transilluminator at room temperature. (1) *Aconitum japonicum* subsp. *subcuneatum* (2) *Anemone flaccida* (3) *Parasenecio delphiniifolius* (4) H_2_O.


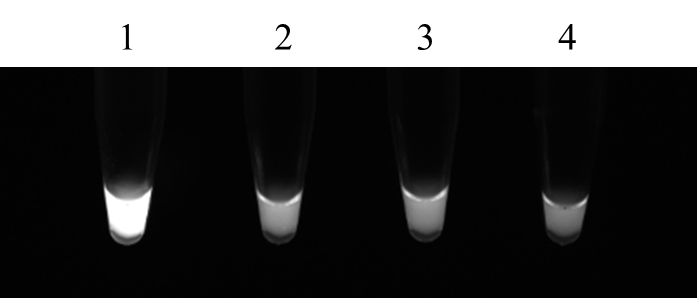

Supplement: Supplementary file 3 — Additional file 3. Sample tubes after LAMP reaction. [file 13104_2019_4463_MOESM3_ESM.docx]
